# Career plateau and career withdrawal intentions among Chinese college physical education teachers

**DOI:** 10.3389/fpsyg.2025.1672181

**Published:** 2025-11-12

**Authors:** Lishan Zhao, Lina Zhao, Huaxing Zhu, Xiaoxia Shang, Cuifeng Gu

**Affiliations:** 1 Physical Education Department, Hebei University of Economics and Business, Shijiazhuang, Hebei Province, China; 2 Physical Education Institute, Hebei Normal University, Shijiazhuang, Hebei Province, China; 3 Department of Materials Engineering, Hebei Construction Material Vocational and Technical College, Qinhuangdao, Hebei Province, China

**Keywords:** job satisfaction, career withdrawal intention, college physical education teacher, career plateau, organizational commitment, mediating effect, moderating effect

## Abstract

**Introduction:**

Examining factors influencing career withdrawal intentions among university physical education (PE) teachers is crucial because of its effects on teaching quality and the educational environment. We examined the effect of career plateauing among college PE teachers in China on their intentions to leave their profession.

**Method:**

A questionnaire survey was conducted with 627 teaching assistants, lecturers, and professors engaged in college PE across Beijing, Tianjin, and Hebei. The underlying mechanisms and boundary conditions were examined, with career satisfaction as a mediating variable and organizational commitment as a moderating variable.

**Results:**

The findings indicated that career plateaus significantly and positively impact career withdrawal intentions by reducing job satisfaction and motivation to remain in the profession. Job satisfaction partially mediates the relationship between career plateaus and career withdrawal intentions. Furthermore, organizational commitment moderates these relationships, shaping the effect of career plateaus on career withdrawal intentions and mitigating the impact of job satisfaction on career withdrawal intentions.

**Discussion:**

This study provides insights into the effect of career plateauing, which is a common challenge affecting PE teachers’ professional development and strengthening their intentions to leave the organization. The findings can inform strategies to retain educators, enhance teaching effectiveness, and improve student experiences.

## 1 Introduction

Physical education (PE) is a crucial component of university education, essential for cultivating comprehensive talent. College PE teachers are the key organizers and executors of PE programs, fostering healthy bodies and high-quality talent. They are among the most critical human resources in universities. In China, while promoting students’ physical and mental health, university teachers face pressures related to self-worth realization, scientific research, and family responsibilities.

In June 2000, China introduced the policy “Implementing Opinions on Deepening the Reform of the Personnel System of Higher Education Institutions,” which proposed an employment system and the elimination of the tenure system (The Central People’s Government of the People’s Republic of China, 2000).

Over the past two decades, most key universities in China have implemented a “non-rise or go” pre-employment system for teachers. However, some local colleges still retain traditional employment structures (Jiang and Wu, 2003). Currently, the profession of PE teachers in Chinese universities is characterized by high skill requirements, intense workloads, and significant pressure (Yuping, 2005). Consequently, some PE teachers take “unpaid leave,” transition to other universities, or resign to pursue different careers. While the intention to leave a job is often termed turnover intention, career withdrawal intention reflects the desire to leave one’s professional field entirely. This deeper disengagement can negatively influence teaching attitudes and behaviors, ultimately impacting students’ learning experiences.

Although extensive research has addressed turnover intentions in China, studies on career withdrawal intentions remain limited, particularly among university PE teachers. The theoretical framework for understanding these intentions still has notable gaps. Given the potential consequences for teaching quality and the educational environment, examining factors influencing career withdrawal intentions among university PE teachers is crucial. Such research can inform strategies to retain educators, enhance teaching effectiveness, and improve student experiences.

Career plateauing is a common challenge in teachers’ professional development. With the growing prevalence of flat organizational structures, vertical progression within hierarchies has become increasingly difficult, leading to career stagnation (Bai et al., 2011). This stagnation often results in reduced productivity, apathy, negative emotions, rebellious attitudes, depression, and turnover, ultimately diminishing individual performance (Rotondo and Perrewé, 2000). Career plateauing also negatively affects organizational effectiveness, reducing commitment and job satisfaction while strengthening individuals’ intentions to leave the organization or withdraw from their careers (Milliman, 1992).

A strong correlation exists between career plateaus, job satisfaction, and organizational commitment in shaping career withdrawal intentions. We investigate how career plateauing among college PE teachers in China affects their intentions to leave their profession. We seek to uncover the mechanisms and boundary conditions of this relationship, using career satisfaction as a mediating variable and organizational commitment as a moderating variable. Additionally, we seek to clarify how the career plateau influences PE teachers’ career withdrawal intentions, so as to provide an empirical basis for improving human resource management at colleges and universities.

## 2 Literature review and hypotheses

### 2.1 Career plateau

Since American psychologist T. P. Ference first proposed the “career plateau” concept in 1977, it has become an important research area in career management. The career plateau refers to the perception that organizational members cannot achieve personal or professional advancement (Tremblay and Roger, 1993). Kou and Zhang (2006) define teachers’ professional plateau as the psychological and behavioral state of temporary stagnation in career progress, such as promotion and mobility, resulting from increased work responsibility at a particular career stage. A review of occupational plateau studies conducted between 1977 and 2017, encompassing 72 empirical sources, revealed that individuals experiencing career plateaus generally report unfavorable outcomes, including reduced satisfaction, diminished well-being, poorer job performance, lower organizational commitment, and heightened turnover intentions (Yang et al., 2019).

### 2.2 Career withdrawal intention

If “wanting to leave the organization” is considered the intention to quit, then the intention of changing one’s career path is “wanting to leave one’s current field of work.” Bedeian et al. (1991) explained the intention of changing work experience as “leaving the current professional field where one fulfills duties and actively seeking positions in other professional fields.” While there are many studies on the intention of quitting, relatively few examine the intention of changing career paths.

### 2.3 Relationship between career plateau and career withdrawal intention

Many scholars have identified the career plateau as a primary factor influencing organizational members’ intentions to change jobs or withdraw from their careers. The greater the degree of career plateau experienced, the stronger the intention to quit (Bedeian et al., 1991). Saleh et al. (2023) highlighted that emotional exhaustion, a consequence of career plateauing, significantly influences early-career employees’ withdrawal intentions. Based on this evidence, the following hypothesis is proposed:

*H*1: The career plateau experienced by college PE teachers strengthens their intention to leave the profession.

### 2.4 Job satisfaction

Locke (1976) defined job satisfaction as “a pleasant or positive emotional state obtained through one’s own evaluation or the position itself.” He and Yao (2009) described the job satisfaction of teachers as a holistic and individually characteristic subjective perception toward their own work and the corresponding work environment.

#### 2.4.1 Career plateau and job satisfaction

Korean scholar Bae Eul-Kyoo demonstrated that when individuals within an organization face career plateaus owing to constraints on promotion, they experience negative consequences such as frustration, reduced work motivation, and diminished job satisfaction (Bae and Kim, 2012). Yee (1990) suggested that teachers’ perceptions of career progression and recognition are integral to their overall job satisfaction, which can be compromised when faced with a sense of stagnation or lack of advancement. This supports the notion that career stagnation negatively affects overall job satisfaction. Based on this, the following hypothesis is proposed:

*H*2: The career plateau among college PE teachers decreases job satisfaction.

#### 2.4.2 Job satisfaction and career withdrawal intention

Numerous studies have confirmed the link between job satisfaction and an individual’s intention to withdraw from their career. In a study of mental health social workers in New York State, Acker (2004) found a strong relationship between job satisfaction and intention to leave. Generally, individuals who feel satisfied with both internal and external aspects of their workplace are less likely to consider leaving. This highlights the connection between job satisfaction and professional withdrawal intentions (Judge and Bono, 2001). Laschinger and Read (2016) highlighted that workplace incivility negatively impacts job satisfaction among new graduate nurses, which in turn strengthens their intention to leave the profession. Based on this evidence, the following hypothesis is proposed:

*H*3: Job satisfaction among college PE teachers weakens their intention to leave the profession.

#### 2.4.3 Intermediary role of job satisfaction

Career stagnation negatively affects organizational effectiveness by reducing members’ job satisfaction, work efficiency, and organizational commitment while increasing turnover and career withdrawal intentions (Milliman, 1992). A career plateau diminishes organizational members’ job satisfaction and engagement, ultimately heightening their intentions to leave (Heilmann et al., 2008). Park (2011) specifically investigated the impact of a career plateau—in terms of both structure and content—on career satisfaction and withdrawal intention among IT service engineers. The findings suggested that experiencing a career plateau negatively affects career satisfaction, which in turn increases the likelihood of withdrawal intentions. For college PE teachers, job satisfaction likely serves as a mediating variable in this relationship. Based on this, the following hypothesis is proposed.

*H*4: Job satisfaction mediates the relationship between the career plateau and career withdrawal intentions for college PE teachers.

### 2.5 Organizational commitment

Porter et al. (1974) introduced the concept of organizational commitment. Their study explored the relationship between organizational commitment, job satisfaction, and employee turnover, laying the groundwork for subsequent research. Organizational commitment refers to an employee’s loyalty and emotional dedication to their company (Porter et al., 1974). This concept encompasses the recognition individuals invest in their organizations, primarily manifesting as psychological attachment and proactive contributions (Francesco and Chen, 2004).

#### 2.5.1 Moderating effect of organizational commitment

Organizational commitment has been linked to career plateaus, job satisfaction, turnover intention, and career withdrawal intention. The roles of career commitment and organizational support in mitigating withdrawal intentions are also evident (Carson et al., 1996; Carson et al., 1997). Carson et al. (1999) consistently found that higher levels of empowerment and organization-based self-esteem are associated with greater job satisfaction and lower withdrawal cognitions. Notably, employees with dual commitment to their organization and career exhibit the highest levels of work satisfaction and the lowest withdrawal intentions, underscoring the protective effect of strong commitment profiles.

Research indicates that experiencing a career plateau, whether hierarchical or content-related, negatively affects organizational commitment and increases the likelihood of employees intending to leave their jobs (Demerouti and Bakker, 2011). Studies have specifically identified hierarchical and job content plateaus as having detrimental effects on occupational commitment (Lin and Chen, 2021).

According to the job demands–resources model, job resources within the workplace play a motivating role, encouraging proactive engagement among employees. Additionally, the interaction between job demands and resources influences employee well-being. Organizational commitment, as a workplace resource, impacts job satisfaction and consequently affects employees’ intentions to leave or withdraw from their careers (Gaither and Mason, 1992). Higher levels of organizational commitment reduce career withdrawal intentions and turnover likelihood (Kusuma and Syah, 2020; Xie and Long, 2008). Based on these findings, the following hypotheses are proposed:

*H*5: Organizational commitment moderates the relationship between career plateaus and job satisfaction among college PE teachers.

*H*6: Organizational commitment moderates the relationship between career plateaus and intention to leave the profession among college PE teachers.

*H*7: Organizational commitment moderates the relationship between job satisfaction and intention to leave the profession among college PE teachers.

In summary, numerous scholars have examined the interplay between career plateaus, job satisfaction, organizational commitment, and career withdrawal intention across various fields, including in teacher-focused studies. However, there have been few studies on college PE teachers in China and other countries. We address this gap by using Chinese college PE teachers as the focal group. Additionally, we incorporate job satisfaction as a mediating variable and organizational commitment as a moderating variable to examine mechanisms and boundary conditions more comprehensively. Through this approach, we aim to provide a deeper understanding of how career plateaus influence career withdrawal intentions among PE teachers by offering targeted recommendations.

## 3 Methods

### 3.1 Sample

We conducted a survey with teaching assistants, lecturers, and professors engaged in college PE teaching in the Beijing, Tianjin, and Hebei regions. Recruitment was carried out through online promotion and offline invitation. We initially planned to recruit 650 people; ultimately, 627 valid responses were obtained (overall response rate: 96.46%). The response rates of the sample schools were balanced, and they can be considered representative of the target group. In China, the “Beijing–Tianjin–Hebei integration” strategy has been elevated to a national priority, with coordinated education development forming a key aspect of regional collaboration. As a vital component of the education system, PE teachers in colleges and universities play a significant role in advancing collaborative education development. Additionally, the concentration of PE teachers in this region offers substantial talent resources for research. Table 1 shows the sample profile. The study was conducted in accordance with the Declaration of Helsinki and approved by the Hebei University of Economics and Business Institutional Review Board. Written informed consent was obtained from all participants.

**Table 1:** Sample overview.

Item	Category	Frequency	Proportion (%)
Gender	Women	299	47.7
	Men	328	52.3
Record of formal schooling	High school and below	6	1.0
	Special course	6	1.0
	Regular college course	183	29.2
	Postgraduate	374	59.6
	Doctoral students	58	9.2
Age (years)	20–30	94	15.0
	31–40	258	41.1
	41–50	200	31.9
	> 50	75	12.0
Professional title	Assistant	93	14.8
	Lecturer	297	47.4
	Associate professor	199	31.7
	Professor	38	6.1
Total		627	100

### 3.2 Questionnaire design and content

The questionnaire comprised two sections. The first section included basic demographics, such as gender, age, education, and professional title. The second section consisted of scales addressing key aspects related to college PE teachers: career plateau, job satisfaction, organizational commitment, and career withdrawal intention.

We utilized previously validated scales commonly used in related research, revised and rephrased to ensure content relevance, representativeness, and item clarity. The Career Plateau Scale for college PE instructors was based on the framework developed by Milliman (1992), which incorporates Bardwick’s structural and content plateau theory. This framework employs a two-dimensional approach to assess the psychological perception of career plateaus. It includes a total of 10 questions: for example, “I think the opportunities for promotion this organization has offered me have reached their limit.” The job satisfaction scale for college PE teachers was derived from the Minnesota Satisfaction Questionnaire (Weiss et al., 1967). This 10-item scale consists of two dimensions: internal and external satisfaction: for example, “I can feel a sense of achievement from my current job.” The organizational commitment scale was based on the framework established by Meyer and Allen (1991). This section of the scale consists of 15 questions: for example, “I have a strong sense of belonging to my current workplace.” Blau (1985) framework provided the foundation for a four-item scale measuring college PE teachers’ intentions to withdraw from their profession: for example, “As a lifelong career, this profession is not ideal.” All questionnaire items were scored on a 5-point Likert scale ranging from “strongly disagree” (1) to “strongly agree” (5), with higher scores reflecting stronger agreement. In all scales, the scores are calculated by averaging the values.

### 3.3 Questionnaire reliability and validity

Confirmatory factor analysis showed that all standardized factor loadings exceeded 0.50. The chi-square to degrees of freedom ratio (x^2^/df) was 3.994, within the acceptable range of 3.00–5.00. The RMSEA value was 0.069, indicating a good fit (0.05–0.08). Fit indices were NFI = 0.929, IFI = 0.946, CFI = 0.946, GFI = 0.898, and TLI = 0.938, all above the 0.90 threshold. These results indicated a strong alignment between the model and data.

Reliability was assessed through internal consistency and composite reliability. Cronbach’s *α* coefficient evaluated internal consistency, and composite reliability was represented by the critical ratio value. Cronbach’s α coefficients for all four dimensions were above 0.7, confirming good internal consistency reliability. Additionally, critical ratio values for each dimension exceeded 0.7, indicating strong composite reliability. The questionnaire’s overall scales and subscales, including Factor 1 (career plateau), Factor 2 (job satisfaction), Factor 3 (organizational commitment), and Factor 4 (career withdrawal intention), demonstrated high reliability.

Validity testing involved both discriminant and convergent validity assessments. Convergent validity, assessed using average variance extracted (AVE), showed values greater than 0.50 for all dimensions, confirming strong convergent validity (Table 2).

**Table 2:** Model AVE, CR index, and Cronbach’s α coefficient results.

Variable	Cronbach’s α	AVE	CR
Factor 1	0.873	0.587	0.876
Factor 2	0.933	0.743	0.935
Factor 3	0.910	0.603	0.913
Factor 4	0.892	0.684	0.895

Factor 1 (career plateau), Factor 2 (job satisfaction), Factor 3 (organizational commitment), and Factor 4 (career withdrawal intention). AVE, average variance extracted; CR, critical ratio.

Discriminant validity was evaluated by comparing AVE values with the correlation coefficients for each dimension. The AVE for each dimension exceeded its correlation coefficients, indicating strong discriminant validity (Table 3).

**Table 3:** Pearson correlation coefficient and AVE square root value.

Variable	Factor 1	Factor 2	Factor 3	Factor 4	AVE
Factor 1	**0.766**				0.587
Factor 2	−0.763^**^	**0.788**			0.743
Factor 3	−0.606^**^	0.761^**^	**0.823**		0.603
Factor 4	0.280^**^	−0.203^**^	−0.162^**^	**0.857**	0.684

The diagonal number is the square root value of AVE. ^**^p < 0.01. Factor 1 (career plateau), Factor 2 (job satisfaction), Factor 3 (organizational commitment), and Factor 4 (career withdrawal intention), demonstrated high reliability. AVE, average variance extracted. The bolded value in the diagonal is the square root of the AVE value.

## 4 Results

### 4.1 Correlation analysis

Pearson’s correlation coefficient was used to assess relationships among variables, with mean values calculated for each dimension. As shown in Table 2, the results indicated significant correlations among the variables (*p* < 0.05), justifying further regression path analysis.

### 4.2 Hypotheses testing

#### 4.2.1 Impact of career plateau on career withdrawal intentions: mediating role of job satisfaction

**Figure 1:**
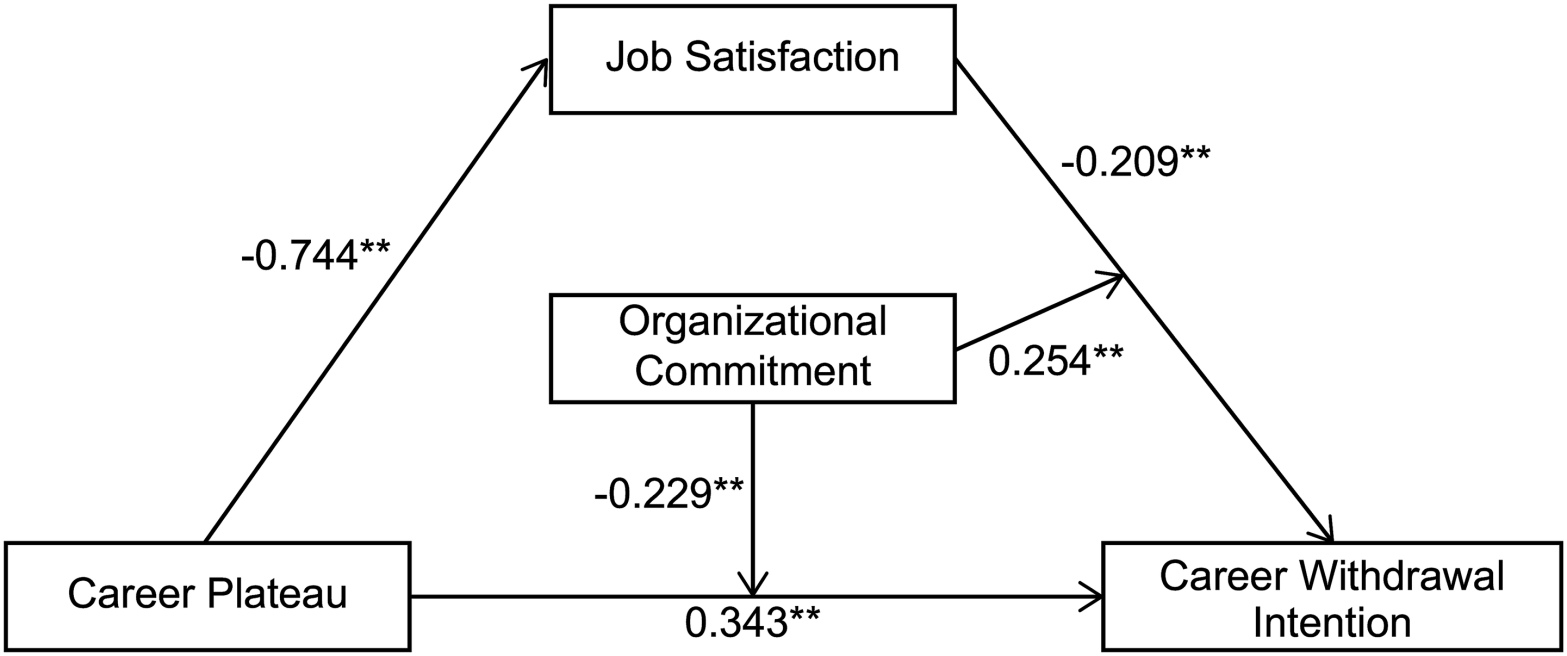
Mediating effect model of job satisfaction among college physical education teachers.

Career plateau was set as the independent variable, career withdrawal intention as the dependent variable, and job satisfaction as the mediating variable. Three models were tested for the mediating effect. In Model 1, we conducted regression analysis between career plateau and career withdrawal intention, determining the total effect (value c). In Model 2, we analyzed the relationship between career plateau and job satisfaction, identifying the intermediate effect (value a). In Model 3, we examined the relationship between career plateau, job satisfaction, and career withdrawal intention, providing insights into the mediating role of job satisfaction. Figure 1 shows the mediating effect model of college PE teachers’ job satisfaction.

Table 4 presents the results. The value of the total effect of career plateau on career withdrawal intention (c) was 0.343, which was significant (*p* < 0.001), indicating that the career plateau experienced by college PE teachers significantly strengthened their intention to withdraw from their careers, thus confirming H1.

**Table 4:** Test table of model with mediating effect of job satisfaction.

Variable	Model 1				Model 2				Model 3			
	*B*	SE	*T*	*Β*	*B*	SE	*t*	*β*	*B*	SE	*t*	*β*
Constant	2.081***	0.104	20.053	-	5.015***	0.059	85.002	-	3.128***	0.366	8.559	-
Career plateau	0.343***	0.050	6.884	0.265	−0.744***	0.028	−26.275	−0.724	0.188***	0.072	2.612	0.145
Job satisfaction									−0.209***	0.070	−2.987	−0.166
*R* ^2^	0.070				0.525					0.084		
Adjusted *R*^2^	0.069				0.524					0.081		
*F*	*F* (1,625) = 47.395, *p <* 0.001				*F* (1,625) = 690.353, *p <* 0.001					*F* (2,624) = 28.458, *p <* 0.001		

The dependent variable in Models 1 and 3 is career withdrawal intention, while the dependent variable in Model 2 is job satisfaction. *p < 0.05; **p < 0.01; ***p < 0.001; SE, standard error.

The impact of career plateau on job satisfaction (a) was −0.744, which was statistically significant (*p* < 0.001), confirming H2. This indicates that the career plateau significantly reduced job satisfaction among college PE teachers.

The effect of job satisfaction on career withdrawal intention (b) was −0.209, which was statistically significant (*p* < 0.001), confirming H3. This indicates that higher job satisfaction among college PE teachers significantly weakened their intention to withdraw from their careers.

After analyzing the results, it was concluded that the career plateau in Model 1 significantly affected job satisfaction (a). Job satisfaction in Model 2 significantly affected career withdrawal intention (b). The career plateau in Model 3 significantly impacted career withdrawal intention (c’), and * a and c’ were equal, indicating a partial mediating role.

In this model, job satisfaction partially mediated the relationship between career plateau and career withdrawal intention. The mediating effect was (−0.744 * −0.209)/0.343 = 45.309%. Thus, career plateau directly and negatively predicted PE teachers’ intention to quit their careers and indirectly predicted this intention through job satisfaction, confirming H4.

#### 4.2.2 Moderating effect of organizational commitment

##### 4.2.2.1 Career plateau–job satisfaction relationship

After standardizing all core variables, the moderating effects were analyzed. Tables 4, 5 provide comprehensive data, and Model 1 indicates that career plateau negatively affected job satisfaction. In Model 4, job satisfaction was the dependent variable, career plateau was the independent variable, and organizational commitment was the moderating variable. The table shows no significant interaction between career plateau and organizational commitment (B = 0.037, *p* = 0.374 > 0.05). This indicates that organizational commitment did not influence the relationship between career plateau and job satisfaction. Therefore, H5 was not supported.

**Table 5:** Test table of model with organizational commitment as a moderating variable.

Variable	Model 4			Model 5			Model 6		
	*B*	SE	*T*	*B*	SE	*t*	*B*	SE	*t*
Constant	3.571***	0.020	175.644	2.724***	0.036	76.578	2.705***	0.035	76.817
Career plateau *organizational commitment	0.037	0.041	0.890	−0.229**	0.072	−3.175			
Job satisfaction *organizational commitment							0.254***	0.063	4.004
*R* ^2^	0.580			0.187			0.243		
Adjusted *R*^2^	0.578			0.183			0.239		
*F*	*F* (3,623) = 286.456, *p <* 0.001			*F* (3,623) = 47.718, *p <* 0.001			*F* (3,623) = 66.596, *p <* 0.001		
△R^2^	0.001			0.013			0.019		
△*F*	*F* (1,623) = 0.793, *p* = 0.374			*F* (1,623) = 10.083, *p* = 0.002			*F* (1,623) = 16.031, *p <* 0.001		

The dependent variable in Model 1 is job satisfaction, while the dependent variable in Models 2 and 3 is career withdrawal intention. *p < 0.05; **p < 0.01; ***p < 0.001.

##### 4.2.2.2 Career plateau–career withdrawal intention relationship

Model 2 indicates that career plateau positively influenced career withdrawal intention. In Model 5, career withdrawal intention was the dependent variable, career plateau was the independent variable, and organizational commitment was the moderating variable. Table 6 illustrates a significant interaction between career plateau and organizational commitment (B = −0.229, *p* < 0.05), suggesting that organizational commitment influenced the relationship between career plateau and career withdrawal intention, thus supporting H6.

**Table 6:** Simple slope analysis of regulation 1.

Adjust variable level	*Β*	SE	*T*	*p*	95% CI	
Mean value	0.436	0.049	8.837	< 0.001	0.339	0.532
High level (+1SD)	0.313	0.068	4.641	< 0.001	0.181	0.446
Low level (-1SD)	0.558	0.057	9.766	< 0.001	0.446	0.670

SE, standard error; CI, confidence interval; SD, standard deviation.

**Figure 2:**
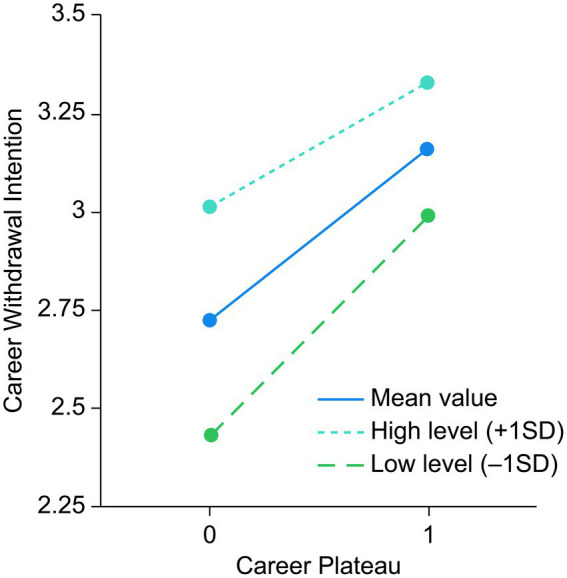
Moderating effect of organizational commitment in the relationship between career plateau and career withdrawal intention.

To further examine the moderating role of organizational commitment among college PE teachers in the relationship between career plateau and career withdrawal intention, a simple slope analysis was conducted. Organizational commitment was divided into high-level (M + 1SD) and low-level (M−1SD) groups to assess how career plateau influenced career withdrawal intention at different commitment levels. The results indicated that, within the low-level group, career plateau significantly predicted the intention to seek change (*β* = −0.558, *t* = 9.766, *p* < 0.001). In the high-level group, while career plateau significantly predicted career withdrawal intention, the effect was weaker (*β* = 0.313, *t* = 4.641, *p* < 0.001). This suggests that as organizational commitment increased, the positive effect of career plateau on withdrawal intention diminished. Figure 2 shows the moderating effect of organizational commitment in the relationship between career plateau and career withdrawal intention.

##### 4.2.2.3 Job satisfaction**–**career withdrawal intention relationship

Model 3 indicated that job satisfaction negatively affected career withdrawal intention. In Model 6, career withdrawal intention was the dependent variable, job satisfaction was the independent variable, and organizational commitment was the moderating variable. Table 7 illustrates a significant interaction between job satisfaction and organizational commitment (*β* = 0.254, *p* < 0.01), suggesting that organizational commitment moderated the relationship between job satisfaction and career withdrawal intention, supporting H7.

**Table 7:** Simple slope analysis of regulation 2.

Adjust variable level	*Β*	SE	*t*	*p*	95% CI	
Mean value	−0.547	0.049	−11.218	< 0.001	−0.642	−0.451
High level (+1SD)	−0.411	0.062	−6.585	< 0.001	−0.534	−0.289
Low level (-1SD)	−0.682	0.056	−12.171	< 0.001	−0.792	−0.572

SE, standard error; CI, confidence interval; SD, standard deviation.

**Figure 3:**
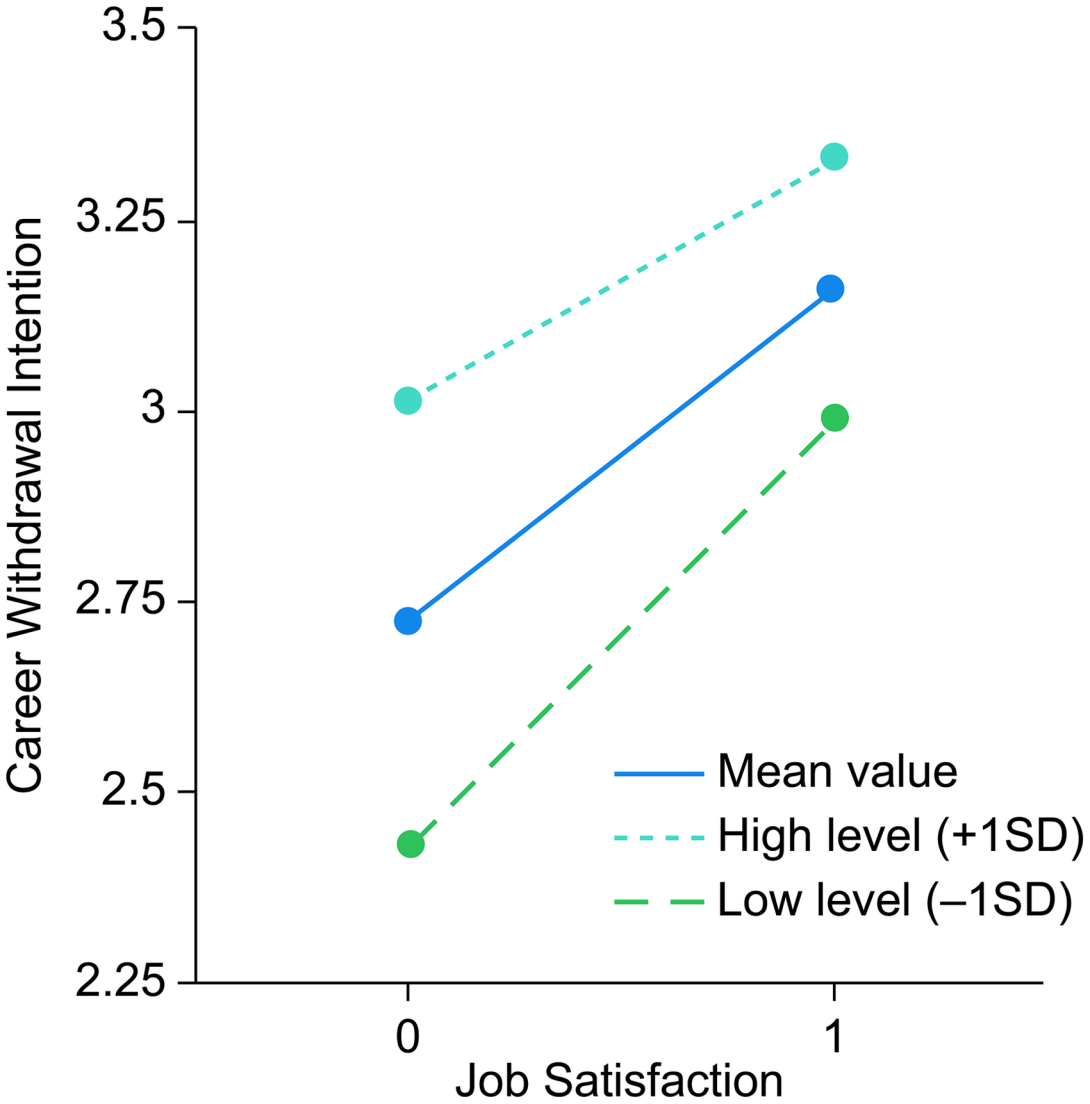
Moderating effect of organizational commitment in the relationship between job satisfaction and career withdrawal intention.

**Figure 4:**
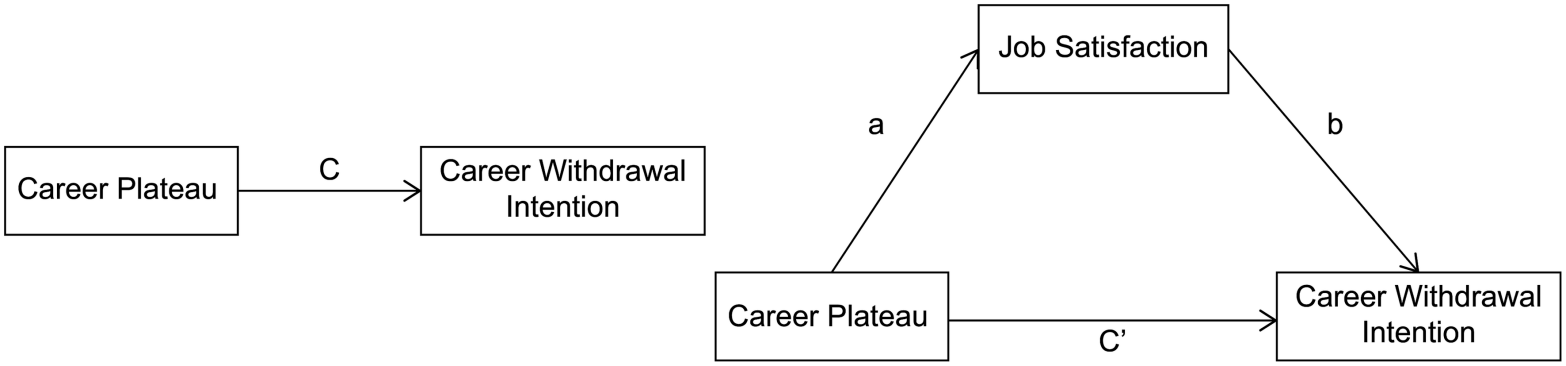
Revised study model showing significant relationships.

To further investigate the moderating role of organizational commitment, a simple slope analysis was conducted. Organizational commitment was categorized into high-level (M + 1SD) and low-level (M−1SD) groups to assess how job satisfaction influenced career withdrawal intention across different commitment levels. The results showed that job satisfaction significantly reduced the likelihood of career withdrawal intention in the low-level group (*β* = −0.682, *t* = 12.171, *p* < 0.001). In the high-level group, while job satisfaction still negatively predicted career withdrawal intention, the effect was weaker (*β* = −0.411, *t* = −6.585, *p* < 0.001). This suggests that as organizational commitment increased, the negative impact of job satisfaction on career withdrawal intention diminished. Figure 3 shows the moderating effect of organizational commitment in the relationship between job satisfaction and career withdrawal intention. Figure 4 shows the model diagram of the results.

## 5 Discussion

### 5.1 Influence of career plateau on career withdrawal intention

We examined how the career plateau experienced by college PE teachers in China affects their career withdrawal intentions, with career satisfaction as a mediating variable and organizational commitment as a moderating variable. The results indicate that a career plateau significantly predicts the career withdrawal intentions of Chinese college PE teachers, confirming H1. This finding aligns with prior research, indicating that a career plateau, whether structural or content-based, negatively influences employees’ perceptions of their organization and significantly increases turnover intentions or the desire to leave the profession (Jin, 2012).

Prolonged exposure to a career plateau may lead individuals to seek alternative career paths (Liang et al., 2016). This phenomenon may be especially complex for college PE teachers in China. Owing to the unique nature of their discipline, PE teachers may face more significant pressure related to professional title promotion and academic achievement. Additionally, as education reforms progress and student needs diversify, PE teachers must continually update their teaching concepts and methods to align with the evolving educational landscape. These factors may aggravate their career plateau and strengthen their intention to withdraw.

### 5.2 Influence of job satisfaction on career withdrawal intention

Job satisfaction negatively predicted the career withdrawal intention of PE teachers in Chinese universities, confirming H2. Numerous scholars have explored the relationship between job satisfaction and career exit intentions among sports facility staff, showing that high job satisfaction generally reduces the likelihood of career transitions (Lee, 2005). Lee et al. (2017) investigated early-career employees and found that job satisfaction significantly affected their turnover intentions, indicating that dissatisfaction during initial career stages can lead to career withdrawal. Pressley and Garside (2023) identified job satisfaction as a key determinant of nurses’ intentions to stay, with variations across generations influencing retention outcomes. Overall, the literature consistently indicates that higher job satisfaction is associated with weaker career withdrawal intentions. Teachers with high job satisfaction are more devoted to their work, investing their time and energy in teaching and pursuing higher teaching quality and academic performance. This positive attitude helps weaken the intention of career withdrawal.

### 5.3 Influence of career plateau on job satisfaction

The findings suggested that a career plateau adversely affected job satisfaction among PE teachers at Chinese universities, supporting H3. When college sports workers perceive limited or no future promotion opportunities, their job satisfaction decreases (Drucker-Godard et al., 2015). The career plateau limits opportunities for career advancement, hindering promotions and higher-level positions. This stagnation reduces job satisfaction and enthusiasm (Judge and Bretz, 1992). During the plateau period, the challenge of balancing responsibilities—such as teaching, research, and administration—often results in role ambiguity, making it difficult to find purpose or fulfillment in work, thereby diminishing job satisfaction. Additionally, PE teachers often receive less societal recognition compared with their peers in other disciplines. This perception of undervaluation exacerbates the difficulties of the plateau period, leading teachers to feel their contributions are neither respected nor adequately rewarded, further eroding job satisfaction (Luo, 2010).

### 5.4 Intermediary role of job satisfaction

The results showed that career satisfaction played an important mediating role in the relationship between career plateau and career withdrawal intention. Career plateaus affect withdrawal intentions indirectly by lowering job satisfaction, which in turn increases the desire to leave. Experiencing a career plateau can diminish motivation and satisfaction among college PE teachers, potentially leading to negative emotions such as stress, anxiety, and depression, which further diminish overall career satisfaction. A lack of career satisfaction, in turn, heightens the career exit intention. When teachers face a career plateau, their job satisfaction decreases owing to limited development opportunities and psychological frustration. This decline in job satisfaction intensifies their inclination to quit. Xie et al. (2016) explored how a career plateau affects turnover intention among employees at the career establishment stage. Their findings suggest that job satisfaction mediates this relationship, indicating that employees experiencing a career plateau tend to have lower job satisfaction, which in turn makes it more likely for them to consider leaving their jobs. Additionally, Noerchoidah et al. (2023) provided evidence that a career plateau influences turnover intention through mediators such as affective commitment and career satisfaction. Their study on hotel employees in East Java demonstrated that perceived stagnation directly affects withdrawal intentions, but that this relationship can be mediated by employees’ emotional attachment and satisfaction with their careers. These findings reinforce the notion that career satisfaction plays an important mediating role in the relationship between career plateau and career withdrawal intention.

### 5.5 Moderating effect of organizational commitment

Three key relationships illustrated how organizational commitment moderated the interplay between career plateau, job satisfaction, and career withdrawal intention. First, organizational commitment did not moderate the relationship between career plateau and job satisfaction among college PE teachers.

Second, organizational commitment moderated the relationship between career plateau and career withdrawal intention. As organizational commitment increased, the positive effect of career plateau on career withdrawal intention decreased. High organizational commitment can help mitigate the negative impact of a career plateau on employees’ intentions to withdraw from their careers. Somers and Birnbaum (2000) identified that employees exhibiting high levels of affective commitment, particularly those committed to both their organization and career, tend to demonstrate more positive work attitudes and a stronger desire to remain with their organization. This suggests that organizational commitment can serve as a protective factor against career withdrawal intentions. Employees who exhibit strong loyalty and recognition toward their organization are more likely to maintain high motivation and engagement when faced with a career plateau, reducing their likelihood of quitting. Conversely, a lack of loyalty toward and recognition within an organization can make employees more susceptible to the adverse effects of a career plateau, potentially strengthening their desire to leave their careers (Wendel and Shin, 2016).

Third, organizational commitment moderated the effect of job satisfaction on career withdrawal intention. As such commitment increased, the negative effect of job satisfaction on career withdrawal intention diminished. Employees committed to their organizations often feel a deeper connection and loyalty, aligning their career growth with the organizations’ progress. Therefore, even with low job satisfaction, they may be less inclined to quit owing to their strong attachment to their organizations (Meyer and Allen, 1991). Employees with high organizational commitment may also alleviate dissatisfaction by adjusting their mindset, seeking internal solutions, or finding alternative sources of satisfaction, thus reducing their intention to leave their careers. Igbaria and Siegel (1992) developed an integrated model of turnover propensity among engineers, incorporating organizational commitment as a predictor alongside job satisfaction. Their findings suggested that organizational commitment provides moderate support in explaining the link between job satisfaction and turnover intentions, indicating that higher commitment can buffer the negative effects of low job satisfaction on career withdrawal. Further, Dewi and Nurhayati (2021) examined how career development influences turnover intention, with organizational commitment serving as a mediating variable. Career development positively affected organizational commitment, which in turn reduced turnover intention, highlighting the moderating and mediating roles of commitment in the satisfaction–withdrawal relationship.

### 5.6 Limitations

Although this study provides valuable insights into the relationship between career plateau, job satisfaction, and career withdrawal intention among college PE teachers, there are several limitations that should be addressed in future research to further enrich our understanding of these dynamics. We adopted a cross-sectional design, gathering data from participants over a relatively short period. A longitudinal follow-up survey was not conducted. Additionally, the sample size could be expanded to enhance representativeness. We used primarily quantitative methods; surveying occupational withdrawal intention is a private topic for college PE teachers, and questionnaire surveys can only provide data without fully capturing descriptive information. Future studies could incorporate interviews to conduct a comprehensive analysis, combining both quantitative and qualitative methods to enhance the accuracy of the findings.

### 5.7 Theoretical implications

This study fills the research gap on career plateau theory in the context of college PE teachers. Previously, this theory was mainly applied in the fields of enterprises and healthcare. However, college PE teachers have both academic and skill-based attributes. We clearly examined the impact of the career plateau on career withdrawal intention. At the same time, we revealed the mediating role of career satisfaction, improving the career plateau–career withdrawal intention theoretical chain. We also verified the moderating effect of organizational commitment, breaking the linear cognition and clarifying the boundary conditions. Moreover, we integrated multiple theories to construct a multivariate model, promoting cross-theory integration and providing a theoretical framework and method reference for multivariate research in the field of educational management.

### 5.8 Practical implications

This study provides theoretical support for Chinese universities in formulating strategies for retaining PE teachers. The results clarify that optimizing career paths, improving incentives, and strengthening organizational culture helps alleviate the problem of teacher attrition. For educational administrative departments, this study offers a basis for optimizing the sports education ecosystem, assisting in the formulation of special policies, resource integration, and the establishment of an exit warning mechanism, thereby addressing regional teacher development issues. For individual teachers, this study guides them to view career plateaus rationally, clarifying that they can achieve breakthroughs by enhancing organizational commitment and independently planning based on their strengths, ultimately achieving a stable team, improving teaching quality and student experience, and balancing teacher development with educational goals.

## 6 Conclusion

This study yielded the following key findings. First, for college PE teachers, career plateauing exerts a significant positive effect on career withdrawal intention—the stronger the perception of career plateauing, the stronger the teachers’ intention to leave their profession.

Second, career plateauing among college PE teachers is significantly and negatively associated with job satisfaction: as teachers perceive more severe career plateauing, their satisfaction with work tends to decrease.

Third, college PE teachers’ job satisfaction shows a significant negative impact on career withdrawal intention—higher levels of job satisfaction correspond to weaker intentions to withdraw from the teaching profession.

Fourth, job satisfaction plays a partial mediating role in the relationship between career plateauing and career withdrawal intention. Specifically, career plateauing not only directly enhances teachers’ career withdrawal intention but also indirectly strengthens this intention by reducing their job satisfaction.

Fifth, organizational commitment moderates the direct effect of career plateauing on career withdrawal intention: the positive effect of career plateauing on career withdrawal intention weakens as organizational commitment increases. In other words, for teachers with high organizational commitment, the impact of career plateauing on their willingness to leave is less pronounced.

Sixth, organizational commitment also moderates the effect of job satisfaction on career withdrawal intention: the negative effect of job satisfaction on career withdrawal intention diminishes with higher organizational commitment. Even when job satisfaction is low, teachers with strong organizational commitment are less likely to develop career withdrawal intention compared with those with low organizational commitment.

## Data Availability

The datasets presented in this article are not readily available because of ethical and privacy restrictions but are available from the corresponding author on reasonable request. Requests to access the datasets should be directed to Lishan Zhao, shan8409@hueb.edu.cn.
